# The anatomy of a data transfer agreement for health research

**DOI:** 10.3389/fphar.2024.1332700

**Published:** 2024-08-27

**Authors:** Lee Swales, Amy Gooden, Donrich Thaldar

**Affiliations:** ^1^ School of Law, University of KwaZulu-Natal, Durban, South Africa; ^2^ Petrie-Flom Center for Health Law Policy, Biotechnology, and Bioethics, Harvard Law School, Cambridge, MA, United States

**Keywords:** data, data transfer agreement, research, scoping review, South Africa

## Abstract

In a data-driven era, the exchange and safeguarding of personal information has become paramount. Data transfer agreements (DTAs) serve to guard privacy, defining the rules for sharing and protecting sensitive data. Yet, the complexities surrounding issues such as data privacy, intellectual property, and dispute resolution within these agreements pose challenges that demand careful consideration. Through a scoping review of twenty-four publicly available, English language DTAs relevant to health research, this article undertakes a comprehensive analysis, examining common clauses, their vital components, and charting a course for responsible data sharing through the provision of insights and practical guidance for drafting DTAs. The article underscores the need for attention to detail and an understanding of data protection legislation in order to ensure that DTAs align with the law and maximize legal certainty.

## 1 Introduction

In a scientific and research context, the transfer of personal information has become routine. One of the key tools used in data protection compliance, and as part of a holistic data management strategy, is a data transfer agreement (DTA). Lawfully managing and strategically sharing data will arguably become more important than it is at present, where already, for at least the past decade, society has recognized that data has value, and the mantra: “*data is the new oil*” has become an oft-repeated line (Parkins, 2017; Swales, 2022). For example, the proliferation of artificial intelligence technologies, such as OpenAI’s *ChatGPT*, that rely on data to produce meaningful output, has further fast-tracked discussions around data transfer, ownership, and management. Additionally, techniques and strategies relating to sharing data are evolving rapidly and should always recognize the value in *some* scientific and academic output. Even where data that does not contain personal information is shared—and, as such, data protection legislation will not apply—it is imperative that this is done intentionally and, in all circumstances, with an eye on the legal consequences (and with consideration for its ownership and value). In most scenarios where data is shared with others, it should be done via a DTA or similar instrument.

What is a DTA? It is a written agreement that facilitates the lawful transfer of data between parties. Typically, an agreement of this type will seek to comply with applicable legislation. Additionally, a DTA will regulate other important legal issues such as ownership of data, intellectual property, the terms of the agreement and how it will terminate, liability, dispute resolution, and whether any consideration is payable (Swales et al., 2023a).

This article presents an empirical study of twenty-four DTAs relevant to health research, which were examined to identify the specific clauses contained therein to tease out key trends and differences. This scoping review facilitates the main part of this article—an *anatomy*, or dissection, of a DTA, where we examine key features of this type of agreement, and make recommendations on critical inclusions together with insight on why these clauses are necessary. This novel scoping review will animate parts of our discussion and assist in providing the guidance set out herein. Accordingly, the purpose of this contribution is to provide academics, researchers, scientists, ethicists, research managers, and all interested stakeholders with guidance on steps to take prior to executing a DTA, and insight into what to include in their own DTAs. Each case will no doubt have nuances and turn on its own facts. To be clear: There is no “one size fits all” template that can be uniformly applied without thought. However, there are many elements of a DTA that will be similar, and the holistic purpose of this discussion is to identify typical features of such an agreement, review best practice, and make recommendations for stakeholders going forward.

## 2 Scoping review

### 2.1 Methodology and results

Although there is no one standard DTA template and the contents of each will depend on certain factors, there are several clauses common to many DTAs. In order to ascertain and understand the key clauses used in DTAs that are contained in the health research space, we conducted a scoping review of publicly available and English-language DTAs. The search terms used are recorded in Supplementary Material 1. The inclusion criterion was that a DTA had to relate to data generally (and hence be inclusive of health research), or specific to health research. To ensure a broad, yet manageable spread of samples, our aim was to collect at least twenty samples, and to ensure that there are at least two samples from the Global South. When we reached twenty-four samples (B3 Africa, 2018; Bristol Myers Squibb, 2017; Clinical Study Data Request Consortium, 2015; Department of Health Western Australia, 2021; Dkfz German Cancer Research Center, 2020; FDP, 2017; Fred Hutch, 2020; GREGoR Consortium, 2022; Growing Up in New Zealand, 2014; Health Data Coalition, 2017; Human Cell Atlas, 2019; Indian Society of Critical Care Medicine, 2024; Infectious Diseases Data Observatory, 2021; Information Commissioner’s Office, 2022; Johns Hopkins University, 2022; Kawartha Lakes OHT, 2020; KEMRI Wellcome Trust Research Programme, 2019; National Center for Advancing Translational Sciences, 2021; National Institute for Medical Research, 2020; National Health Service England, 2018; ONDC, 2024; Swiss Personalised Health Network, 2021; University of Newcastle, 2024; Utrecht University, 2024) containing two samples from Africa (B3 Africa, 2018; KEMRI Wellcome Trust Research Programme, 2019) and one from India (Indian Society of Critical Care Medicine, 2024), we decided that we had reached saturation point. Each DTA was examined to identify the specific clauses contained therein. The most frequently occurring clauses across the twenty-four DTAs were identified. These clauses were tabulated and categorized to facilitate a comprehensive comparison (see Supplementary Material 2). To gain insights into the prevalence and consistency of these clauses, their content was compared across all DTAs. Through this comparison, the common features shared by the clauses were identified—and encompassed language, structure, and substantive content of the provisions. The common clauses that we found were:

• Introduction (preamble/recitals), definitions, and parties.
• Purpose.
• Term and termination.
• Obligations on parties.
• Reporting and auditing.
• Intellectual property (and licensing).
• Data ownership.
• Publication and attribution.
• Confidentiality.
• Limitation of liability.
• General provisions (or miscellaneous).
• Governing law.
• Dispute resolution.

### 2.2 Limitations

Our study does have limitations. First, the sample size of twenty-four DTAs, while broad and representative of five continents, was intentionally kept to a size that we perceived as manageable. Second, our scoping review was confined to agreements available in the English language and freely accessible online. As such, the results may not fully capture the global landscape of DTAs. A further caveat is that terminology and definitions that are used in DTAs may vary across jurisdictions, and that the substantive provisions found in DTAs may cater for specific institutional needs or reflect domestic (national) legal requirements. Nevertheless, we suggest that the results of our scoping exercise are informative and useful. In the next section, we discuss the results in more detail.

## 3 Discussion: key features of a data transfer agreement

### 3.1 Introduction, definitions, and parties

Most commercial agreements begin with an introduction, also known as a preamble, or recitals (also referred to as “whereas” clauses). Like any good story or piece of writing, the introduction provides exactly that: An introduction to what is about to come. As Murray (2018) points out, this clause identifies the “who, what, when, and why” in the agreement. As noted by an English court in Toomey Motors v Chevrolet (2017), the fact that this clause is introductory in nature, does not mean its provisions are not binding, and these clauses may contain “operative provisions.”

However, it is a matter of style and personal preference in deciding which clause comes first, and the order that follows. One might also see a definitions clause coming first, and that clause being followed by the introductory clause. The definitions clause is usually accompanied by an interpretation clause. This is a technical legal clause that provides a list of definitions and legal interpretative clauses. Usually, words used in the agreement with a capital letter will be defined terms and will be included in the definitions list—a definition is included in the agreement to assist with flow, and to aid the reader. For example, if a word or term has a long and/or complicated meaning, it is usually included in the definitions list (for example, “Intellectual Property” or “Processing Purpose”).

Typically, near the start of the agreement, there is a clause that fully describes the parties to the agreement. This, as is the case with many parts of a contract, can be achieved in a multitude of ways: A clause on its own, or as part of the introduction, included in the definitions, or even on the cover page.

Given the importance of introductions and explanations, most of the DTAs that we analyzed included some form of introduction, definitions, and information about the parties—although these did vary depending on the DTA. Some provided an introduction or background (Bristol Myers Squibb, 2017; Clinical Study Data Request Consortium, 2015; Department of Health Western Australia, 2021; Fred Hutch, 2020; Growing Up in New Zealand, 2014; Health Data Coalition, 2017; Human Cell Atlas, 2019; Infectious Diseases Data Observatory, 2021; Information Commissioner’s Office, 2022; National Center for Advancing Translational Sciences, 2021; ONDC, 2024; University of Newcastle, 2024), while others contained a recital (Dkfz German Cancer Research Center, 2020; Fred Hutch, 2020; Kawartha Lakes OHT, 2020; National Institute for Medical Research, 2020; Swiss Personalised Health Network, 2021; Indian Society of Critical Care Medicine, 2024). Some of the DTAs included a definitions section (Bristol Myers Squibb, 2017; Clinical Study Data Request Consortium, 2015; Department of Health Western Australia, 2021; Dkfz German Cancer Research Center, 2020; Fred Hutch, 2020; GREGoR Consortium, 2022; Growing Up in New Zealand, 2014; Health Data Coalition, 2017; Human Cell Atlas, 2019; Indian Society of Critical Care Medicine, 2024; Infectious Diseases Data Observatory, 2021; Kawartha Lakes OHT, 2020; KEMRI Wellcome Trust Research Programme, 2019; National Institute for Medical Research, 2020; Swiss Personalised Health Network, 2021; Utrecht University, 2024.), although in some it appeared as an appendix or glossary (GREGoR Consortium, 2022; Information Commissioner’s Office, 2022; National Health Service England, 2018). All twenty-four DTAs provided information about the parties or a blank space in which information could be added.

### 3.2 Purpose

A purpose clause sets out the primary intention of the parties and articulates the nature of the agreement. This clause provides additional context, and sets out rights, responsibilities, and restrictions. In the context of a DTA, it is important to record the data transfer, the reason for the transfer, and note any important restrictions and obligations on the parties.

Ten of the DTAs that we examined contained a specific purpose clause (Department of Health Western Australia, 2021; Dkfz German Cancer Research Center, 2020; Human Cell Atlas, 2019; Indian Society of Critical Care Medicine, 2024; Information Commissioner’s Office, 2022; Kawartha Lakes OHT, 2020; National Health Service England, 2018; ONDC, 2024; Swiss Personalised Health Network, 2021; University of Newcastle, 2024). Although some DTAs may not have a specific “purpose” clause, information relevant to the purpose was nevertheless included in other clauses (B3 Africa, 2018; Bristol Myers Squibb, 2017; Clinical Study Data Request Consortium, 2015; FDP, 2017; Fred Hutch, 2020; GREGoR Consortium, 2022; Health Data Coalition, 2017; Infectious Diseases Data Observatory, 2021; Johns Hopkins University, 2022; KEMRI Wellcome Trust Research Programme, 2019; National Center for Advancing Translational Sciences, 2021; National Institute for Medical Research, 2020; Utrecht University, 2024).

There is significant variance in how narrow or broad, general or detailed purpose statements are formulated, which may be a reflection of the legal tradition in the relevant jurisdiction. This is demonstrated by the following example:

Improvements in information sharing, translate into many tangible benefits. Repeat diagnostic tests can be avoided. Medical errors are reduced and outcomes improved with quicker access to complete information. Time is saved by physicians, staff and patients. With less manual processing of information and fewer phone calls for results, patients can be cared for quicker.

Ultimately patients will be more engaged in their care by leveraging the technology where providers and patients can securely access necessary PHI.

Participants may include hospitals, healthcare organizations and healthcare providers involved in the circle of care that or who have direct involvement in the delivery of patient care, which requires the communication and sharing of patient information.

This data sharing agreement is entered into by the Participants to enable more effective and efficient patient information sharing that then will translate into better patient care (Kawartha Lakes OHT, 2020).

By contrast, the University of Newcastle (2024) DTA contains a checklist of the various purposes for which the data is to be used:

The Data is to be used only for the study of eating behaviours. Please indicate from the options below how you intend to use the Data:

• Training and evaluation of new machine learning models for the detection of eating behaviours

• Benchmarking existing machine learning models for the detection of eating behaviours

• Creating and/or analyzing metrics of eating behaviors (e.g., eating pace and duration)

• Other. Please specify (University of Newcastle, 2024):

An insightful drafting note is included in the National Health Service England (2018) template agreement in the purpose clause to assist those that use the template (the advice should be heeded in developing any purpose clause). An excerpt of it is below:

Document the detail to explain the purpose and objectives of the information sharing … ensure that all parties affected by the information sharing are clear about why the information may be used… National Health Service England (2018)

We suggest that all the purposes of the sharing should be listed. It should be made clear which organization is processing the data and for which purpose. It is important to specify this in sufficient detail and exactness, as DTAs typically limit the processing of the data by the recipient to the defined purpose. In other words, should the data recipient process the data for any purpose other than the defined purpose, it would be in breach of contract.

### 3.3 Term and termination

An important feature of any agreement is its term, and the manner of its termination. One must also be aware of the agreement’s effective date (the date the agreement is binding from). With a DTA, usually there is a fixed term, with the ability for either party to give notice to the other to terminate (also known as cancellation for convenience—or no-fault termination—where one party does not need to give a reason for termination). Linked to this clause, one will usually also see a termination for fault or cause (a breach clause), and a clause which sets out how termination for convenience should be achieved. Some of the DTAs that we analyzed dealt with term and termination under one clause (Clinical Study Data Request Consortium, 2015; Bristol Myers Squibb, 2017; Fred Hutch, 2020; Infectious Diseases Data Observatory, 2021; Indian Society of Critical Care Medicine, 2024), while others either dealt with term and termination separately, or combined them with another clause (B3 Africa, 2018; Department of Health Western Australia, 2021; Dkfz German Cancer Research Center, 2020; FDP, 2017; GREGoR Consortium, 2022; Growing Up in New Zealand, 2014; Health Data Coalition, 2017; Human Cell Atlas, 2019; Information Commissioner’s Office, 2022; Johns Hopkins University, 2022; Kawartha Lakes OHT, 2020; KEMRI Wellcome Trust Research Programme, 2019; National Center for Advancing Translational Sciences, 2021; National Institute for Medical Research, 2020; National Health Service England, 2018; Swiss Personalised Health Network, 2021; University of Newcastle, 2024; Utrecht University, 2024).

Above all, parties should know: (1) when the agreement is effective from; (2) how long it lasts for; and (3) how they can terminate the agreement, and under what circumstances. Below are two examples of this type of clause:

Term and Termination. 22.1 This Agreement shall be effective as of the Effective Date and, unless cancelled or terminated earlier in accordance with the terms hereof, shall continue in effect until 30 September 2002 (the “Initial Term”). Thereafter, this Agreement shall continue in force and effect unless and until cancelled or terminated as provided in this Agreement (Law Insider, 2024).

Termination for Convenience. Either party may terminate this Agreement without cause and at any time upon giving 30 days’ prior written notice to the other party (each, a termination for “Convenience”). Such termination will be effective on the date stated in the notice (NetDocuments, 2024).

The first example displays a fixed term agreement clause where the agreement comes to an end on a specific date. Parties would also be able to terminate for cause on the basis of a clause found elsewhere in that agreement. The second example shows a termination for convenience clause where either party can terminate the agreement on notice without any fault and without having to give a reason. This type of clause provides maximum flexibility. Typically, where research institutions are involved, for the protection of both parties, one would want to see a termination for convenience clause so that a party is not forced to stay in a relationship that does not suit it. However, there may be economic or other factors that require the contract to exist for a long period, and for no termination for convenience to exist. Each case will turn on its own facts and this is a point parties must consider carefully.

Below are two examples of term and termination clauses found in the DTAs that we examined:

7.1. This Agreement shall come into force on the Effective Date and will remain in effect for a period of one (01) year from the Effective Date or on the expiration of a thirty (30) days’ written notice by either party.

7.2. This Agreement will terminate immediately upon any breach of the provisions of this Agreement by the Recipient or by any of the Registered Users.

7.3. In the event that this Agreement is terminated in accordance with this Clause 7.1 or 7.2, the Recipient shall return or destroy all Data at the direction of the Provider (Indian Society of Critical Care Medicine, 2024).

And:

This Agreement will expire on the completion of the Research and completion of the publications included in the Publication Plan but in no event later than three (3) years from the Effective Date. BMS may terminate this Agreement for Institution’s material breach of its terms, where the breach is not cured within thirty (30) days following receipt of written notice of same. Upon termination or expiration of this Agreement the rights and obligations of the Parties which have accrued hereunder shall survive in accordance with their terms, and Institution’s right to use BMS Confidential Information shall immediately cease. The terms of Section 3 (Term and Termination), 4 (Institution Representations, Warranties and Covenants), 5 (Confidentiality), 6 (Publication), 7 (Inventions), 8 (Miscellaneous) shall survive the expiration or termination of this Agreement (Bristol Myers Squibb, 2017).

### 3.4 Obligations on parties

The clause (or clauses) that set out the main obligations of the parties can be drafted in many ways, and different headings can be used. Twenty-one of the DTAs that we examined contained a clause (or information) detailing the obligations or duties of the parties to the agreement (B3 Africa, 2018; Clinical Study Data Request Consortium, 2015; Department of Health Western Australia, 2021; Dkfz German Cancer Research Center, 2020; FDP, 2017; Fred Hutch, 2020; GREGoR Consortium, 2022; Growing Up in New Zealand, 2014; Health Data Coalition, 2017; Human Cell Atlas, 2019; Infectious Diseases Data Observatory, 2021; Information Commissioner’s Office, 2022; Johns Hopkins University, 2022; Kawartha Lakes OHT, 2020; KEMRI Wellcome Trust Research Programme, 2019; National Center for Advancing Translational Sciences, 2021; National Institute for Medical Research, 2020; National Health Service England, 2018; Swiss Personalised Health Network, 2021; University of Newcastle, 2024; Utrecht University, 2024).

Below is an excerpt of a DTA clause which lists the obligations (we have only reproduced part of the clause because of its length) of the parties:

It is hereby agreed that the following conditions to the Agreement shall be binding on the RECIPIENT:

(a) The RECIPIENT agrees to use, store or dispose of the DATA in compliance with all applicable laws including those relating to research involving the use of human and animal subjects.

(b) The DATA shall remain the property of the PROVIDER and PROVIDER hereby consents to the DATA being made available as a service to the research community.

(c) The RECIPIENT shall use the DATA for teaching or academic research purposes only.

It is hereby agreed that the following conditions to the Agreement shall be binding on the PROVIDER:

(a) The PROVIDER agrees to transfer, store or dispose of the DATA in compliance with all applicable laws

(b) The PROVIDER shall transfer immediately the DATA upon receipt of one of the two copies duly signed by the RECIPIENT (National Institute for Medical Research, 2020).

This clause creates contractual obligations (or duties) on both parties. Usually, one would expect to find the key responsibilities of the parties in this clause. In the context of a DTA, primarily, one should ensure the clause places obligations on the parties to comply with the conditions of lawful processing set out in South Africa’s Protection of Personal Information Act 4 of 2013 (POPIA), 2013 (or equivalent international legislation). As can be seen in the example above, both the provider and recipient have a duty to ensure compliance with “all applicable laws”—one could craft this to specifically refer to data protection legislation, such as POPIA.

Typically, one would also see obligations on the parties in relation to dealing with data after the relationship ends (in other words, to return or delete it), and in terms of how to *use* the data (such as for teaching or academic research purposes only). If there are specific requirements or nuances to a project, this is the clause that will list those requirements. We suggest that parties give careful thought to what the project entails—simply put, what is it each party needs to do in order to achieve a successful outcome, and then to ensure these obligations are listed in this clause.

Holistically, we suggest that a DTA can be a useful tool to facilitate compliance with data protection legislation. In this context, parties may consider including provisions that relate to the following:

• The ground of justification for the transfer;
• The manner in which the data was collected, how it will be processed, transferred, stored, and disposed of;
• Data subject access rights;
• Appropriate technical and organizational measures are taken, and that adequate safeguards are in place;
• Measures in place in relation to cross border data flows;
• Conditions and restrictions in place in relation to further processing of data beyond.

Parties should also ensure that the details and mechanics of the data being transferred are included in the agreement. As all of the agreements that we examined are DTAs, they all mention the transfer of data in some form. However, not all DTAs described the mechanics of such transfers (Growing Up in New Zealand, 2014; Clinical Study Data Request Consortium, 2015; Bristol Myers Squibb, 2017; Health Data Coalition, 2017; National Health Service England, 2018; KEMRI Wellcome Trust Research Programme, 2019; Dkfz German Cancer Research Center, 2020; Kawartha Lakes OHT, 2020; Department of Health Western Australia, 2021; Indian Society of Critical Care Medicine, 2024; University of Newcastle, 2024; Utrecht University, 2024). Twelve DTAs provided more detailed guidance relating to transfers of data (B3 Africa, 2018; FDP, 2017; Fred Hutch, 2020; GREGoR Consortium, 2022; Human Cell Atlas, 2019; Infectious Diseases Data Observatory, 2021; Information Commissioner’s Office, 2022; Johns Hopkins University, 2022; National Center for Advancing Translational Sciences, 2021; National Institute for Medical Research, 2020; ONDC, 2024; Swiss Personalised Health Network, 2021). For practical reasons, this could be an annexure. Only six DTAs provided for the transfer of data in an annexure (FDP, 2017; Human Cell Atlas, 2019; Fred Hutch, 2020; National Institute for Medical Research, 2020; Swiss Personalised Health Network, 2021; Johns Hopkins University, 2022).

### 3.5 Reporting and auditing

An example of an a-typical clause in a DTA relates to auditing and reporting. Only two of the DTAs in our scoping review contained an audit clause (Growing Up in New Zealand, 2014; Kawartha Lakes OHT, 2020), which appear as follows:

The Privacy Officer of each Participant shall audit access to PHI for which the Participant is the Custodian, including without limitation access by its Authorized Users (Kawartha Lakes OHT, 2020).

And:

A representative of UniServices will be permitted access by the Institution, at all reasonable times, to the results and analyses obtained from the use of the Data Set together with any records and documents relating thereto for the purpose of verifying compliance with the conditions of this Agreement. The Institution will provide UniServices with any information which UniServices reasonably requests in relation to the Institution’s compliance with this Agreement (Growing Up in New Zealand, 2014).

The primary purpose of a clause such as this is to allow the provider to ensure that the recipient is taking adequate steps to comply with its obligations. Despite the importance of this clause, very few of the DTAs that we analyzed contained specific clauses relevant to reporting and auditing (Growing Up in New Zealand, 2014; Kawartha Lakes OHT, 2020; Infectious Diseases Data Observatory, 2021). None of the DTAs examined contained a specific reporting clause, and in eight of the DTAs reporting is instead mentioned either generally throughout the agreement or under another clause (Health Data Coalition, 2017; National Health Service England, 2018; Kawartha Lakes OHT, 2020; Infectious Diseases Data Observatory, 2021; Swiss Personalised Health Network, 2021; GREGoR Consortium, 2022; Indian Society of Critical Care Medicine, 2024; University of Newcastle, 2024).

One will also see clauses that require one party to report to the other in relation to, for example, processing activities with the data, and safeguards in place—and in some cases this type of obligation may be found in the main obligations clause discussed above in 3.4. Parties should consider what best suits their needs in the context of the data involved. However, we do suggest parties should have some ability to assess whether the other party is complying with the agreement.

### 3.6 Intellectual property and licensing

A specific intellectual property (IP) clause was present in twelve of the DTAs that we examined (B3 Africa, 2018; Clinical Study Data Request Consortium, 2015; Department of Health Western Australia, 2021; Dkfz German Cancer Research Center, 2020; Fred Hutch, 2020; Growing Up in New Zealand, 2014; Human Cell Atlas, 2019; Indian Society of Critical Care Medicine, 2024; Infectious Diseases Data Observatory, 2021; Swiss Personalised Health Network, 2021; University of Newcastle, 2024; Utrecht University, 2024), with another four dealing with IP under other clauses (Bristol Myers Squibb, 2017; KEMRI Wellcome Trust Research Programme, 2019; Kawartha Lakes OHT, 2020; National Institute for Medical Research, 2020).

IP clauses are like Janus, with one face looking back and one face looking forward. It looks back in the sense that it recognizes pre-existing IP rights, often termed as “background” IP. It also looks forward, and provides for rights in any new IP that is created by the Recipient using the Project Data. Typically, the Recipient will own the IP that it creates using the Project Data, but this can be negotiated. For example, the Recipient can grant a perpetual nontransferable use-license to the Provider in the IP that it creates, or the parties can be joint owners of the IP. Here is an example of a simple IP clause:

Except for the rights explicitly granted hereunder, nothing contained in this Agreement shall be construed as conveying any rights under any patents or other intellectual property which either Party may have or may hereafter obtain (Human Cell Atlas, 2019).

Licensing is often dealt with under the IP clause. Ten of the DTAs from our scoping review include licensing within IP (Clinical Study Data Request Consortium, 2015; Bristol Myers Squibb, 2017; Human Cell Atlas, 2019; Dkfz German Cancer Research Center, 2020; Fred Hutch, 2020; Department of Health Western Australia, 2021; Infectious Diseases Data Observatory, 2021; Swiss Personalised Health Network, 2021; Indian Society of Critical Care Medicine, 2024; University of Newcastle, 2024). Examples of licensing provisions (within an IP clause) is as follows:

Provider grants to Recipient the non-exclusive, worldwide, perpetual, sub-licensable, royalty-free, fully paid up license to use all Data for Recipient’s non-commercial, research and educational purposes (Indian Society of Critical Care Medicine, 2024).

And:

Subject to any pre-existing rights, obligations, options to license, or licenses granted by the Provider and/or Recipient to a third party, the Recipient and Provider retain or are granted a non-exclusive royalty-free license to use an Invention developed under the Purpose for their own research, educational, patient care purposes but not for Commercial Use unless otherwise outlined in the Implementing Letter (Fred Hutch, 2020).

Licensing is mentioned in relation to ownership as well as commercialization, as can be seen below:

The University grants the Recipient Organisation a non-exclusive, non-transferable, fee-free licence to use the Data for the Purpose only.

If the Recipient Organisation wishes to commercialise or have commercialised any Results or Data IP, or otherwise deal in the Data or Derivatives for any commercial purpose, it must first enter into an appropriate licence agreement with the University (University of Newcastle, 2024).

Next, we consider data ownership. It is important to note that although both data ownership and IP pertain to incorporeal objects, data ownership and IP are distinct legal concepts and are governed by different legal rules.

### 3.7 Data ownership

The Project Data would presumably consist of one or more computer files—i.e., digital objects. Each of these digital objects has an independent existence in the digital world, has value and usefulness, and can be controlled by humans. As such, in legal systems that have a basis in Roman Law, the Project Data should be susceptible of being owned (Thaldar et al., 2022). Yet, data ownership remains controversial in the West. By contrast, China is leading the way with the adoption of a policy on the commercialization of data, released in 2022 (Xiong et al., 2023). This policy provides for various property rights modules in data. If the data contains personal information, a privacy module applies to the data in addition to the property rights modules. With China officially endorsing data as legal property, we suggest that it would be unwise for the rest of the world to remain in data ownership purgatory.

It is essential to address and dispel the primary objection to data ownership, especially concerning personal data. This argument is structured as follows:

• Premise 1: In certain situations, the ownership rights of a data generator (like a university) might conflict with the privacy rights of data subjects.
• Premise 2: Political and legal policies underscore the importance of data privacy rights, as evidenced by the growing body of global legislation on the matter.
• Conclusion: Therefore, data ownership is viewed as politically and legally untenable.

While the premises are true, the conclusion does not necessarily hold. Thaldar et al. (2022) argue that ownership is always encumbered in some way, depending on the nature of the object and the circumstances. In the context of personal data, ownership is encumbered by privacy rights, allowing for a reconciliation between data ownership and data privacy. This perspective aligns with China’s approach that provides that if data is personal data, the property rights in such data are superseded by the privacy rights of the data subjects. In a recent article, Thaldar (2024) turns the anti-data-ownership argument on its head by showing that research institutions can only properly fulfil their statutory duties to protect the personal data in their care if they actively claim ownership in such data. Thaldar (2024) uses an example of a person who has lawful access to the data at a research institution, such as a research collaborator or a student, who makes a copy of the file containing the relevant data on her own memory stick and deletes the original file from the research institution’s system. Subsequently, the person declares herself the owner of the data contained in the file on the memory stick. If the research institution shunned data ownership, it has none of the well-established civil and criminal remedies of an owner available. It will have to rely on its contractual relationship with the person who took the data, which places it in a significantly weaker position. As Thaldar (2024) concludes, data ownership is a precondition for being an effective data custodian.

In agreements like DTAs, we propose that while ensuring the protection of individuals’ data privacy rights through contractual obligations is crucial, as discussed above under Section 3.4, it is equally important to explicitly articulate ownership rights. This dual focus can harmonize the protection of privacy with the recognition of data as a valuable and ownable asset.

Let’s now consider the results of the scoping review. Sixteen of the DTAs that are part of our scoping review mention “ownership.” However, on closer inspection, only six of these DTAs unambiguously provide for *data ownership*—i.e., where the object of ownership is *data per se*, as distinct from *rights in data*, such as IP rights in data (B3 Africa, 2018; Human Cell Atlas, 2019; Indian Society of Critical Care Medicine, 2024; National Institute for Medical Research, 2020; Swiss Personalised Health Network, 2021; University of Newcastle, 2024). This is an important distinction. Claiming only IP rights in data and remaining silent about the data itself, means that ownership of the data itself—which is independent of any IP rights in the data—remains unresolved. Yet, this is the case in the majority of the DTAs that we reviewed. Two DTAs even conflate the objects of ownership (Dkfz German Cancer Research Center, 2020; Utrecht University, 2024). For example, one DTA provides:

The RECIPIENT recognizes that nothing in this Agreement shall operate to transfer to the RECIPIENT or its RECIPIENT SCIENTISTs any INTELLECTUAL PROPERTY rights in or relating to the DATA, i.e., ownership of DATA remains unchanged (Dkfz German Cancer Research Center, 2020).

This kind of conceptual confusion should be avoided. A clear data ownership provision, such as the following simple provision should be included in any DTA:

As this is an ISCCM initiated project, the entire ownership of the data will be with the ISCCM (Indian Society of Critical Care Medicine, 2024).

It is important that data ownership exists independently and distinctly from ownership of rights in the data, such as IP rights. As such, it makes sense to deal with these two kinds of objects of ownership in under separate headings. However, it can also be successfully combined in a single clause, provided that the concepts are not conflated, as illustrated by the following provision:

The Receiving Institute will own all Research Data, results, inventions, copyright in datasets, sui generis database rights, and all associated rights, which arise which arise under the Research Project described in Appendix A (Human Cell Atlas, 2019).

An argument that is sometimes heard in academic circles is that because there is legal uncertainty about data ownership in a given jurisdiction, referring to data ownership should best be avoided as a component of a DTA. This argument is mistaken. If there is still a dearth of caselaw on data ownership in a given jurisdiction, resulting in the issue not yet being settled law, this fact is good reason to *explicitly* provide for data ownership in a DTA—in this way, the parties are bound to the agreed position. For example, if a recipient agreed that the provider is the owner of the project data (*qua* well-defined digital object), the recipient could be estopped from later asserting in court that the provider is not the owner. Accordingly, including an explicit data ownership provision in a DTA creates legal certainty—even in an environment of general uncertainty.

### 3.8 Publication and attribution

Typically, in data transfers involving universities or research institutions, one can expect to see a clause regulating the publication of results and/or academic publications. Only one DTA in our scoping review contained a specific attribution clause (Human Cell Atlas, 2019), but thirteen DTAs included publication clauses (B3 Africa, 2018; Bristol Myers Squibb, 2017; Clinical Study Data Request Consortium, 2015; Department of Health Western Australia, 2021; Dkfz German Cancer Research Center, 2020; Fred Hutch, 2020; Health Data Coalition, 2017; Human Cell Atlas, 2019; Indian Society of Critical Care Medicine, 2024; Infectious Diseases Data Observatory, 2021; Swiss Personalised Health Network, 2021; University of Newcastle, 2024; Utrecht University, 2024). In eight of the DTAs, publication was mentioned under another clause (Growing Up in New Zealand, 2014; FDP, 2017; KEMRI Wellcome Trust Research Programme, 2019; National Institute for Medical Research, 2020; National Center for Advancing Translational Sciences, 2021; GREGoR Consortium, 2022; Johns Hopkins University, 2022; ONDC, 2024).

As a starting point, we recommend that no results are released unless the other party consents. However, it is not unusual to expect that the party who provided the data would want the right to stipulate whether or not the results are published, and to retain the right to derive benefit from academic publications.

Further, given obligations imposed by data protection legislation, it is prudent to insert a provision regulating how results are made public. An example may appear as follows:

As SPHN projects are funded with public money, the Parties strive to make the resulting scientific publications publicly accessible and available through Open access as far as possible according to publishers rights (Swiss Personalised Health Network, 2021).

And:

The Receiving Institute must endeavour to publish results in an open access academic journal or database (Human Cell Atlas, 2019).

One would also expect to see something here, including an obligation to make acknowledgments. Fifteen DTAs required acknowledgements to be made in publications arising from the provider’s data (B3 Africa, 2018; Bristol Myers Squibb, 2017; Department of Health Western Australia, 2021; Dkfz German Cancer Research Center, 2020; Fred Hutch, 2020; Growing Up in New Zealand, 2014; Health Data Coalition, 2017; Human Cell Atlas, 2019; Indian Society of Critical Care Medicine, 2024; Infectious Diseases Data Observatory, 2021; KEMRI Wellcome Trust Research Programme, 2019; National Center for Advancing Translational Sciences, 2021; National Institute for Medical Research, 2020; Swiss Personalised Health Network, 2021; University of Newcastle, 2024). An example of such a provision reads as follows:

Publications: Unless directed otherwise, HDC must be acknowledged in any publication or presentation using HDC data, and the following disclaimer must appear on any materials developed for public distribution with data used under this DSA: “The views expressed herein do not necessarily represent the views of HDC (Health Data Coalition, 2017).”

And:

Recipient will acknowledge the Provider as the source of the Data in any publication reporting on its use, unless requested otherwise by the Provider (Indian Society of Critical Care Medicine, 2024).

And:

The Institution will ensure that all outputs that are intended for publication, including (but not necessarily limited to) reports, journal papers, working papers, conference and other public presentations, and other documents, contains an acknowledgement that the Data Set has been sourced from The University of Auckland, Growing Up in New Zealand: Longitudinal Study of New Zealand Children and Families, together with an appropriate acknowledgement of the funders of the study, all of which must be approved by the Data Access Committee in writing prior to the publication (Growing Up in New Zealand, 2014).

### 3.9 Confidentiality

A confidentiality provision is a standard clause in any commercial agreement, and a DTA is no exception. As with any other clause, there are many ways to draft this—typically, the clause stipulates that each party will keep all information (which will be broadly defined) confidential, and will not, without the prior written consent of the other party, disclose to any person any of the confidential information. This prohibition on disclosure of confidential information will usually not preclude any party from making any disclosure to its professional advisors (provided that the advisors ensure the information remains confidential). Further, it will preclude a party from making any disclosure which it is required to make by law (such as in the course of an investigation around a data breach).

The importance of confidentiality can be seen in the fact that thirteen of the DTAs in our scoping review contained a dedicated clause dealing with confidentiality (B3 Africa, 2018; Bristol Myers Squibb, 2017; Clinical Study Data Request Consortium, 2015; Department of Health Western Australia, 2021; Dkfz German Cancer Research Center, 2020; Fred Hutch, 2020; Health Data Coalition, 2017; Human Cell Atlas, 2019; Indian Society of Critical Care Medicine, 2024; Kawartha Lakes OHT, 2020; Swiss Personalised Health Network, 2021; University of Newcastle, 2024; Utrecht University, 2024). An additional five DTAs, although not including a dedicated confidentiality clause, mentioned confidentiality—in some form or another—throughout the DTA (National Health Service England, 2018; KEMRI Wellcome Trust Research Programme, 2019; Infectious Diseases Data Observatory, 2021; National Center for Advancing Translational Sciences, 2021; GREGoR Consortium, 2022). An example of a confidentiality clause is as follows:

Either PARTY shall treat the CONFIDENTIAL INFORMATION confidential for the duration of this Agreement, including any extension thereof, and thereafter for a period of five (5) years following termination or expiry of this Agreement. Excluded from this obligation of confidentiality shall be any CONFIDENTIAL INFORMATION of which one PARTY can reasonably demonstrate that it (a) was previously known to them, or (b) is, and/or becomes, publicly available during said five (5) year period through no fault of a PARTY, or (c) is independently and lawfully developed by one PARTY. This obligation of confidentiality shall not apply to any disclosure required by law, provided that the RECIPIENT shall notify the PROVIDER of any disclosure required by law in sufficient time so that the PROVIDER may contest such requirement, if the PROVIDER so chooses. Subject to mandatory law, upon the expiration or termination of this Agreement for whatever reason, or at the earlier request of a PARTY, the other PARTY shall, at its own costs, return or destroy all originals and copies of CONFIDENTIAL INFORMATION, or, in case of CONFIDENTIAL INFORMATION stored in electronic, magnetic or digital media, shall erase or render unreadable all materials furnished (including without limitation, working papers containing any CONFIDENTIAL INFORMATION or extracts therefrom) which contain CONFIDENTIAL INFORMATION (Swiss Personalised Health Network, 2021).

And—note that Human Cell Atlas (2019) defines “Research Materials” to include, *inter alia*, “Research Data collected for the Research Project”:

8.1. The Information may include confidential information of the Providing Institute. Accordingly, if and to the extent that any such Information is marked as “confidential,” the Receiving Institute shall during the Term of this Agreement and for a period of [*insert period*] following its termination, treat such Information as confidential and only disclose it under like obligations of confidentiality and Restrictions on Use as those contained herein. The Receiving Institute shall be deemed to have fulfilled its obligation if it [*insert local criteria applicable to confidentiality standards/requirements*].

8.2. The above-mentioned obligations of confidentiality shall not apply to Information which:

8.2.1. [*If contributing derived Research Data to the HCA:* Is identified as Research Data to be contributed to the HCA by the Providing Institute/Receiving Institute, as listed in Appendix A]; or

8.2.2. Can be shown to have been known to the Receiving Institute at the time of its acquisition from Providing Institute; or

8.2.3. Is acquired from a third party, not in breach of any confidentiality obligation to the Providing Institute; or

8.2.4. Is independently devised or arrived at by, on behalf of, or for the Receiving Institute without access to the Information; or

8.2.5. Enters the public domain otherwise than by breach of the undertakings set out in this Agreement.

8.3. In some cases, the Research Materials may also incorporate confidential Information pertaining to research participants or donors having provided the Research Materials. The Research Materials provided to the Receiving Institute have been [*enter information related to de-identification processes applied to the data, e.g., coded, double-coded, anonymized, anonymous (provide description of de-identification measures)*]. If the Receiving Institute inadvertently receives Information that identifies individual research participants or donors, the Receiving Institute will take all reasonable and appropriate steps to protect the privacy and confidentiality of such Information. This may require immediate destruction of the Research Materials on request of the Providing Institute. The Receiving Institute agrees to make no intentional attempt to re-identify research participants or donors, through linkage of data or otherwise. The Receiving Institute will immediately report any identification of research participants or donors to the Providing Institute (Human Cell Atlas, 2019).

### 3.10 Limitation of liability

Another of the “boilerplate” clauses (those clauses which you see in almost every commercial agreement, irrespective of what the agreement regulates), is a clause limiting the liability of the parties—sometimes, this may be coupled with indemnities. In larger, more complicated commercial deals these two clauses will be separated, but for purposes of a data transfer, it may well be that one can combine them. Nine of the DTAs in our scoping review contained a liability clause (which is often combined with warranties) (B3 Africa, 2018; Department of Health Western Australia, 2021; Growing Up in New Zealand, 2014; Human Cell Atlas, 2019; Indian Society of Critical Care Medicine, 2024; Information Commissioner’s Office, 2022; Kawartha Lakes OHT, 2020; Swiss Personalised Health Network, 2021; Utrecht University, 2024). In eleven DTAs, liability is mentioned under another clause, such as limitations and exclusions (Infectious Diseases Data Observatory, 2021), data sharing (Clinical Study Data Request Consortium, 2015), disclaimer (GREGoR Consortium, 2022), terms and conditions (FDP, 2017; National Center for Advancing Translational Sciences, 2021; Johns Hopkins University, 2022), indemnification (Fred Hutch, 2020), warranty and indemnities (University of Newcastle, 2024), legal statement (Dkfz German Cancer Research Center, 2020), remedies and no waiver (National Health Service England, 2018), and obligations of provider and recipient (National Institute for Medical Research, 2020).

In terms of the various limitation of liability provisions, for the most part, where the clause exists, it attempts to protect the provider of the data, and ensure that it will not be liable for damages relating from the use or transfer of the data (B3 Africa, 2018; Dkfz German Cancer Research Center, 2020; FDP, 2017; Human Cell Atlas, 2019; Indian Society of Critical Care Medicine, 2024; Infectious Diseases Data Observatory, 2021; Johns Hopkins University, 2022; National Institute for Medical Research, 2020; Utrecht University, 2024).

An example of this clause is as follows:

Providing Institute will not be liable for damages related to the provision of Research Materials to the Receiving Institute. This includes but is not limited to damages in relation to inaccuracies, lack of comprehensiveness, or use of the Research Materials, or any delays or break in supply by the Providing Institute (Human Cell Atlas, 2019).

Interestingly, two of the DTAs required that parties take out, and maintain, liability insurance for the duration of the agreement (Kawartha Lakes OHT, 2020; Department of Health Western Australia, 2021)—with one DTA specifying the value of the insurance (Kawartha Lakes OHT, 2020).

We suggest that the limitations, as far as possible, should be reciprocal, and that both parties indemnify each other from unlawful conduct. Importantly, both parties should identify a figure that represents the entire amount any party could claim from another. The context will determine the appropriate figure, and this will be informed by the level of risk, insurance cost, and benefit derived from the project.

Parties should also ensure that neither party will be liable for loss of profits or consequential damages arising out of the project.

Further examples of liability clauses are as follows:

11.1 Providing Institute makes no warranty, either express or implied, of the fitness for purpose of the Research Material. However, to the best of Providing Institute’s knowledge, the use of the Research Materials within the Purpose of Use shall not infringe on the proprietary rights of any third party.

11.2 Providing Institute will not be liable for damages related to the provision of Research Materials to the Receiving Institute. This includes but is not limited to damages in relation to inaccuracies, lack of comprehensiveness, or use of the Research Materials, or any delays or break in supply by the Providing Institute. The Receiving Institute acknowledges that the Providing Institute makes no guarantee that the Research Materials are free of contamination from viruses, latent viral genomes, or other infectious agents. The Receiving Institute agrees to treat the Research Materials as if they were not free from contamination, to ensure that appropriate biosafety training is provided to research personnel, and to implement appropriate biohazard containment measures.

11.3 The Receiving Institute agrees that, except as may explicitly be provided for in this Agreement, the Providing Institute has no control over the use that is made of the Research Materials or the Information by the Receiving Institute in accordance with the terms of this Agreement. Consequently, the Receiving Institute agrees that Providing Institute shall not be liable for such use.

11.4 The Receiving Institute will not be liable for damages incurred by the Providing Institute in providing the Research Materials to the Receiving Institute. This includes but is not limited to damages incurred through the Providing Institute’s breach of contract or statute, its breach of institutional policy, research ethics requirements, as well as any tortious or extracontractual liability incurred (Human Cell Atlas, 2019).

And:

Except to the extent prohibited by law, the Recipient assumes all liability for damages which may arise from its use, storage, disclosure, or disposal of the Data. The Provider will not be liable to the Recipient for any loss, claim, or demand made by the Recipient, or made against the Recipient by any other party, due to or arising from the use of the Data by the Recipient, except to the extent permitted by law when caused by the gross negligence or willful misconduct of the Provider (FDP, 2017).

And:

6.1 Nothing in this Agreement excludes or limits the liability of either Party:

6.1.1 for death or personal injury caused by that Party’s negligence; or

6.1.2 for fraud or fraudulent misrepresentation; or

6.1.3 to the extent that such liability cannot be limited or excluded by law.

6.2 Subject to Clause 6.1, in no event will the University of Oxford or the Data Contributor(s) be liable for any use of the Dataset by the Recipient, whether in contract, tort (including negligence or breach of statutory duty) or otherwise howsoever arising (Infectious Diseases Data Observatory, 2021).

### 3.11 General provisions (miscellaneous)

Fourteen of the twenty-four DTAs that we examined contained a heading for general provisions, or sometimes called “Miscellaneous” (B3 Africa, 2018; Bristol Myers Squibb, 2017; Department of Health Western Australia, 2021; GREGoR Consortium, 2022; Growing Up in New Zealand, 2014; Health Data Coalition, 2017; Human Cell Atlas, 2019; Indian Society of Critical Care Medicine, 2024; Infectious Diseases Data Observatory, 2021; Information Commissioner’s Office, 2022; National Health Service England, 2018; Swiss Personalised Health Network, 2021; University of Newcastle, 2024; Utrecht University 2024). The general clauses serve as a backbone to the overall contract, addressing various fundamental legal, operational, and administrative aspects that govern the relationship between the parties involved. These clauses are pivotal for ensuring clarity, legality, and fair practice in data transfers. The components (or sub-clauses) commonly found in these clauses are as follows:

• Waiver: This provision clarifies that the failure or delay in enforcing any part of the agreement does not constitute a waiver of rights.
• Assignment and Novation: This provision dictates the conditions under which parties can transfer their rights and obligations under the DTA to another party.
• Relationship of the Parties: It clarifies that the DTA does not create a partnership, joint venture, or agency relationship between the parties.
• Amendment: This specifies that changes to the DTA must be made in writing and signed by all parties.
• Severability: If any part of the DTA is found to be invalid or unenforceable, this provision allows for that part to be removed without affecting the remainder of the DTA.
• Entire Agreement: This provision states that the DTA constitutes the full and complete agreement between the parties, superseding all prior discussions and agreements.

Less common, but very useful components of general clauses are:

• Survival Clause: This provision specifies which provisions of the agreement will continue to be effective after the termination or expiry of the agreement. For example, the Department of Health Western Australia (2021) DTA specifies that certain clauses will survive the termination or expiry of the agreement.
• Counterparts: Some DTAs, like those of National Health Service England (2018), allow the agreement to be executed in counterparts, meaning separate copies can be signed and assembled to form the complete agreement.
• Contact Points and Notices: This provision specifies how formal communications related to the DTA should be made, often requiring written notices, as seen in the University of Newcastle (2024) DTA.
• Electronic Signatures and Form: With the advancement of technology, some DTAs, like the Swiss Personalised Health Network (2021), acknowledge electronic signatures and communications.

Two provisions that are sometimes found as sub-clauses under the general clause, but also frequently as self-standing clauses, are governing law and dispute resolution. We discuss these two provisions next.

### 3.12 Governing law

The inclusion of a governing law provision is a fundamental aspect of a DTA, as it establishes which country’s law will govern the interpretation of the DTA. Typically, a governing law provision will also provide which court within the relevant country has jurisdiction to adjudicate disputes that arise from the DTA. In our analysis, it was observed that almost all the DTAs reviewed incorporate a governing law provision. Only six DTAs (FDP, 2017; KEMRI Wellcome Trust Research Programme, 2019; National Center for Advancing Translational Sciences, 2021; GREGoR Consortium, 2022; Johns Hopkins University, 2022; ONDC, 2024) eschew this essential element.

The DTAs that contain a governing law provision typically specify the country whose law will govern the DTA. However, in two cases, the Human Cell Atlas (2019) and B3 Africa (2018), the choice of jurisdiction is left open for the parties to decide.

Interestingly, among the eighteen DTAs that do include a governing law provision, ten delineate it as an independent clause (Clinical Study Data Request Consortium, 2015; Dkfz German Cancer Research Center, 2020; Fred Hutch, 2020; Human Cell Atlas, 2019; Indian Society of Critical Care Medicine, 2024; Infectious Diseases Data Obervatory, 2021; Information Commissioner’s Office, 2022; Kawartha Lakes OHT, 2020; National Institute for Medical Research, 2020; Swiss Personalised Health Network, 2021), seven integrate it within the general or miscellaneous provisions (B3 Africa, 2018; Bristol Myers Squibb, 2017; Department of Health Western Australia, 2021; Growing Up in New Zealand, 2014; National Health Service England, 2018; University of Newcastle, 2024; Utrecht University 2024), and one defines the governing law under its definitions/interpretations section (Health Data Coalition, 2017). This differentiation in presentation underscores the varied approaches to structuring DTAs.

An example of a governing law clause is found in the Utrecht University (2024) DTA. It reads as follows:

This agreement will be governed by the laws of Netherlands and disputes concerning its execution will be put before the competent district court of Utrecht (Utrecht University, 2024).

We suggest that this concise example is worth emulation. The absence of such a governing law clause means that resolving disputes could become complicated, potentially necessitating judicial intervention to ascertain applicable laws. Such situations could lead to unforeseen legal entanglements and protracted disputes, which could counteract the purpose of the DTA.

### 3.13 Dispute resolution

Most DTAs in our scoping review dealt with dispute resolution in some form. Of the twenty-four DTAs that we examined, four contained a dedicated dispute resolution clause (Growing Up in New Zealand, 2014; National Health Service England, 2018; Human Cell Atlas, 2019; Kawartha Lakes OHT, 2020). Twelve of the DTAs dealt with (or simply mentioned) dispute resolution under another clause (B3 Africa, 2018; Bristol Myers Squibb, 2017; Clinical Study Data Request Consortium, 2015; Department of Health Western Australia, 2021; Dkfz German Cancer Research Center, 2020; Fred Hutch, 2020; Health Data Coalition, 2017; Indian Society of Critical Care Medicine, 2024; Information Commissioner’s Office, 2022; National Institute for Medical Research, 2020; Swiss Personalised Health Network, 2021; Utrecht University, 2024)—most commonly the governing law clause or the general provisions clause. Those DTAs that dealt with disputes under the governing law or general provisions clauses referred to the jurisdiction and the laws that will apply (Bristol Myers Squibb, 2017; Clinical Study Data Request Consortium, 2015; Dkfz German Cancer Research Center, 2020; Indian Society of Critical Care Medicine, 2024; Information Commissioner’s Office, 2022; Swiss Personalised Health Network, 2021; Utrecht University, 2024). Others mentioned alternative dispute resolution mechanisms, such as arbitration, negotiation, and mediation (B3 Africa, 2018; Department of Health Western Australia, 2021; Fred Hutch, 2020; Growing Up in New Zealand, 2014; Health Data Coalition, 2017; Human Cell Atlas, 2019; Indian Society of Critical Care Medicine, 2024; Information Commissioner’s Office, 2022; Kawartha Lakes OHT, 2020; National Institute for Medical Research, 2020; National Health Service England, 2018).

Holistically, it is important to ensure that the clause provides clarity on how a dispute will be managed—and, in our view, a tiered approach is best in this type of relationship. What do we mean by a tiered approach? The parties should be obliged to try and meet first to find a solution to the dispute by negotiation (usually senior representatives from both sides), failing that, a formal mediation, and then an arbitration using well known rules. However, parties should include a provision that acknowledges that either party may be able to approach a court of law on an urgent basis. In some cases, parties may need urgent or interim relief pending the outcome of the negotiations, mediation, or arbitration, and it is wise to ensure that a party is not prevented from seeking such urgent, interim relief.

We also suggest including a provision to stipulate that the mediation or arbitration will be held via video conferencing, unless the parties agree otherwise—this will likely assist from a cost saving perspective, and should also expediate matters. Following the COVID-19 pandemic, video conferencing, such as Zoom and Microsoft Teams, is commonplace.

Ultimately, a dispute resolution clause should provide the parties with an efficient, pragmatic, and cost-effective manner to resolve any dispute. An example of a dispute resolution clause is as follows:

16.1 All disputes arising out of or in connection with this Agreement shall be settled under the Rules of Arbitration of the International Chamber of Commerce by one or more arbitrators appointed in accordance with the said Rules.

16.2 The Parties agree, pursuant to Article 30 (2) (b) of the Rules of Arbitration of the International Chamber of Commerce, that the Expedited Procedure Rules shall apply, provided the amount in dispute does not exceed US$ [specify amount] at the time of the communication referred to in Article 1 (3) of the Expedited Procedure Rules.

16.3 The Parties agree that arbitration shall be conducted in [CITY] at [PLACE].

16.4 Legal proceedings brought by a Party while this Agreement is in force, and legal proceedings brought by a Party arising out of or in connection with this Agreement may only be brought in the courts of [JURISDICTION] at [JUDICIAL DISTRICT]. This clause shall only have effect if, for any reason, a dispute cannot be brought to arbitration pursuant to the preceding clauses (Human Cell Atlas, 2019).

## 4 Conclusion

In a rapidly evolving data-driven landscape, DTAs stand as foundational instruments governing the exchange of data across various sectors, from scientific research to commercial partnerships. This comprehensive analysis sheds light on the critical clauses that underpin DTAs, highlighting their importance in facilitating secure, efficient, and legally compliant data-sharing relationships and providing guidance on the drafting of such clauses. Drafting DTAs requires attention to detail, a nuanced understanding of data protection regulations and, often, legal expertise. DTAs are pivotal, not only for safeguarding data, but also for fostering collaboration, innovation, and responsible data sharing. With the guidance and insights provided in this article, stakeholders can navigate the complexities of data transfers, maximize legal certainty, and adhere to evolving data protection laws.

We should mention that the findings of the scoping review formed the basis for an open-source DTA template that was developed for the South African research community (Swales et al., 2023a; Swales et al., 2023b).
