# Supplementary material for: The anatomy of a data transfer agreement for health research
Source: Front Pharmacol. 2024 Aug 27;15:1332700. doi: 10.3389/fphar.2024.1332700 (PMC11383768; doi:10.3389/fphar.2024.1332700)
Supplement: Supplementary file 1 [file DataSheet1.zip › Supplementary Material 1.docx]

Supplementary Material 1

# Search terms

The following search terms were used in our scoping review (in alphabetical order):

- “data sharing agreement Africa"
- “data sharing agreement America”
- “data sharing agreement Canada”
- “data sharing agreement South America”
- “data transfer agreement Africa”
- “data transfer agreement America”
- “data transfer agreement Europe”
- “data transfer agreement Kenya”
- “data transfer agreement NIH”
- “data transfer agreement pdf”
- “data transfer agreement South Africa”
- “data transfer agreement University of Witwatersrand ”
- “data use agreement America”
- “data use agreement Australia”
- “national health institute”
- “UK biobank data transfer agreement”
- “university data transfer agreement”

# Websites

The following websites were visited as part of our scoping review (in alphabetical order):

- Bristol Myers Squibb (bms.com)
- Clinical Study Data Request Consortium (clinicalstudydatarequest.com)
- Department of health (Western Australia) (rgs.health.wa.gov.au)
- Dkfz German Cancer Research Center (dfkz.de)
- Fred Hutch (research.fredhutch.org)
- GREGoR Consortium (gregorconsortium.org)
- Human Cell Atlas (humancellatlas.org)
- Indian Society of Critical Care Medicine (isccm.org)
- Infectious Diseases Data Observatory (iddo.org)
- Information Commissioner’s Office (ico.org.uk)
- Johns Hopkins University (jhura.jhu.edu)
- Kawartha Lakes Escibe (pub-kawarthalakes.escribemeetings.com)
- Kenyan Medical Research Institute (kemri-wellcome.org)
- National Center for Advancing Translational Sciences
- National Institute for Medical Research (nimr.or.tz)
- NHS England (england.nhs.uk)
- Office of the National Data Commissioner (datacommissioner.gov.au)
- Research Gate (researchgate.net)
- Swiss Personalised Health Network (swissethics.ch)
- University of Auckland (cdn.auckland.ac.nz)
- University of Newcastle, Australia (newcastle.edu.au)
- UT Health San Antonio (uthscsa.edu)
- Utrecht University, Netherlands (uu.nl)

# Data transfer agreements

Below is a list of the 24 DTAs that we obtained in our scoping review (in alphabetical order):

- B3 Africa. (2018). *Data Transfer Agreement*. https://www.researchgate.net/publication/357269852_DATA-TRANSFER-AGREEMENT
- Bristol Myers Squibb. (2017). *Data Sharing Agreement*. https://www.bms.com/assets/bms/us/english-documents/independent-research/sample-data-sharing-agreement.docx
- Clinical Study Data Request Consortium. (2015). *Data Sharing Agreement*. https://www.clinicalstudydatarequest.com/Documents/DATA-SHARING-AGREEMENT.pdf
- Department of Health Western Australia. (2021). *Material and Data Transfer Agreement*. https://rgs.health.wa.gov.au/Documents/Material%20and%20Data%20Transfer%20Agreement%20v1%2012%20Nov%202021.docx
- Dkfz German Cancer Research Center. (2020). *Data Transfer Agreement of Human Data for Research Purposes*. https://www.dkfz.de/en/CanEpi/EGA/Data-Transfer-Agreement-of-Human-Data-for-Research-Purposes-Jan.-2020.pdf
- FDP. (2017). *Data Transfer and Use Agreement*. https://www.uthscsa.edu/sites/default/files/Services/forms/duatemplate.pdf
- Fred Hutch. (2020). *Multi-Party Data and Material Transfer Agreement*. https://research.fredhutch.org/content/dam/stripe/specimen-acquisition-network/COL210703_Umbrella%20Data%20and%20Material%20Transfer%20Agreement_FH_FINAL%20no%20org%20list.pdf
- GREGoR Consortium. (2022). *Data Sharing Agreement*. https://gregorconsortium.org/sites/default/files/policies/GREGoR%20Consortium%20Data%20Sharing%20Agreement%20V1.0_Fillable.pdf
- Growing Up in New Zealand. (2014). *Data Access Agreement*. https://cdn.auckland.ac.nz/assets/growingup/access-to-data/External%20DA%20Agreement_1003a_V2.pdf
- Health Data Coalition. (2017). *Data Sharing Agreement*. https://hdcbc.ca/wp-content/uploads/HDC_Data_Sharing_Agreement_v2.0-4.pdf
- Human Cell Atlas. (2019). *Template: Material/Data Transfer Agreement (MDTA)*. https://docs.google.com/document/d/1MpUXC7ayY7RAYeZrEMbEdoYn2l-rIBDR/edit
- Indian Society of Critical Care Medicine. (n.d.). *Data Transfer Agreement*. https://isccm.org/isccm_v1/pdf/data_transfer_agreement.pdf
- Infectious Diseases Data Observatory. (2021). *COVID-19 Data Platform: Data Transfer Agreement*. https://www.iddo.org/sites/default/files/publication/2021-03/COVID-19%20Data%20Transfer%20Agreement%2016MAR21.pdf
- Information Commissioner’s Office. (2022). *International Data Transfer Agreement*. https://ico.org.uk/media/for-organisations/documents/4019538/international-data-transfer-agreement.pdf
- Johns Hopkins University. (2022). *Data Transfer and Use Agreement*. https://jhura.jhu.edu/wp-content/uploads/2022/03/DUATemplate_Updated.pdf
- Kawartha Lakes OHT. (2020). *Data Sharing Agreement*. https://pub-kawarthalakes.escribemeetings.com/filestream.ashx?DocumentId=37434
- KEMRI Wellcome Trust Research Programme. (2019). *Application Form for Access to KWTRP Data*. https://kemri-wellcome.org/zp-content/uploads/2021/02/KWTRP_Dataverse_Data_Request_Form_2019.docx
- National Center for Advancing Translational Sciences. (2021). *Data Transfer Agreement*. https://ncats.nih.gov/files/NCATS_Data_Transfer_Agreement_508.pdf
- National Health Service (NHS) England. (2018). *Template Data Sharing Agreement*. https://www.england.nhs.uk/wp-content/uploads/2019/04/appendix-4-sample-data-sharing-agreement.docx
- National Institute for Medical Research. (2020). *Data Transfer Agreement for Researchers/Organizations*. http://reims.nimr.or.tz:8010/guides/DTA.pdf
- ONDC. (n.d.). *Data Sharing Agreement*. https://www.datacommissioner.gov.au/sites/default/files/2022-07/ONDC_Legislation_Agnostic_DSA_Template.doc
- Swiss Personalised Health Network. (2021). *Data Transfer and Use Agreement*. https://sphn.ch/wp-content/uploads/2021/11/2018DEV21_DTUA_SPHN_SwissPKcdw_V3.0.pdf
- University of Newcastle. (n.d.). *Data Transfer Agreement*. https://www.newcastle.edu.au/__data/assets/pdf_file/0010/631954/Data_Transfer_Agreement_OREBA1.pdf
- Utrecht University. (n.d.). *Data Transfer Agreement*. https://www.uu.nl/sites/default/files/data_transfer_agreement_youth_template.pdf
