# Supplementary material for: The anatomy of a data transfer agreement for health research
Source: Front Pharmacol. 2024 Aug 27;15:1332700. doi: 10.3389/fphar.2024.1332700 (PMC11383768; doi:10.3389/fphar.2024.1332700)
Supplement: Supplementary file 1 [file DataSheet1.zip › Supplementary Material 2.docx]

Supplementary Material 2

# Introduction

Below is the table of results from the scoping review. Each data transfer agreement (DTA) is included, as well as the common identified clauses and whether they were present in each DTA.

| **DTA** | **Introduction, preamble, recital, definitions, and parties** | **Purpose** | **Term and termination** | **Obligations on parties** | **Reporting and auditing** | **Intellectual property** | **Data ownership** | **License to use data** | **Publication and attribution** | **Dispute resolution** | **Confidentiality** | **Limitation of liability** | **General provisions (miscellaneous)** | **Governing law** |
| --- | --- | --- | --- | --- | --- | --- | --- | --- | --- | --- | --- | --- | --- | --- |
| **Human Cell Atlas**  ***Material/Data Transfer Agreement*** | ✓  Contains information about the parties, an introduction, and a definitions clause. | ✓  Contains a clause on purpose(s) of use. Purpose is also present in the introduction. | ✓  Contains a clause on term of the agreement and a clause on amendment, extension, termination, and survival. | ✓  No specific obligations clause, but obligations are mentioned throughout. | X  No reporting or auditing clause. | ✓  Contains a clause on intellectual property. | ✓  Ownership is dealt with under the clause on intellectual property. | ✓  Licensing is dealt with under the clause on intellectual property. | ✓  Contains a clause on attribution. Also contains a clause on research data and publications. | ✓  Contains a clause on dispute resolution. | ✓  Contains a confidentiality clause. | ✓  Contains a clause on warranties and liabilities. | ✓  Contains a miscellaneous clause. | ✓  Contains a governing law clause. |
| **Infectious Diseases Data Observatory**  ***COVID-19 Data Platform: Data Transfer Agreement*** | ✓  Contains a background clause, as well as information about the parties, and a definitions clause. | ✓  Purpose is indirectly dealt with under the background clause. Additionally, purpose is covered under the data transfer clause and the recipient obligations clause. | ✓  Contains a duration and termination clause. | ✓  Contains a recipient obligations clause. | ✓  Contains a clause on reporting and intellectual property. | ✓  Contains a clause on reporting and intellectual property. | ✓  Ownership is dealt with under the clause on reporting and intellectual property. | ✓  No specific clause on licensing, but licensing is mentioned under obligations, reporting and intellectual property, and duration and termination. | ✓  Contains a publication clause. | X  No dispute resolution clause. | X  No confidentiality clause (and only confidential information is mentioned). | ✓  Liability is dealt with under the limitations and exclusions clause. | ✓  Contains a general provision clause. | ✓  Contains a governing law clause. |
| **B3 Africa**  ***Data Transfer Agreement*** | X  No introduction or definitions clauses. However, there is information about the parties. | ✓  No specific purpose clause, but there is a clause on authorized use of the data which deals with purpose. | ✓  Term and termination is dealt with under the warranties and liability clause (and termination is further dealt with under the miscellaneous clause). | ✓  Contains a clause dealing with the agreement between the parties. | X  No clauses on reporting and auditing (or any mention of it). | ✓  Contains a clause on intellectual property and ownership. | ✓  Contains a clause on intellectual property and ownership. | X  No licensing clause (or any mention of licensing). | ✓  Contains a publications clause. | ✓  Disputes are dealt with under the miscellaneous clause. | ✓  Contains a confidentiality clause. | ✓  Contains a clause on warranties and liability. | ✓  Contains a miscellaneous clause. | ✓  No governing law clause (mentioned under the miscellaneous clause). |
| **FDP**  ***Data Transfer and Use Agreement*** | X  No introduction or definitions are provided, but there is space for party details. | ✓  No specific purpose clause, but purpose is mentioned. | ✓  No specific termination clause, but deals with termination. | ✓  No specific obligations clause, but the duties of the parties are provided. | X  No reporting or auditing clause. | X  No intellectual property clause (or any mention of intellectual property). | ✓  No ownership clause, but ownership is mentioned under the terms and conditions. | X  No licensing clause (or any mention of licensing). | ✓  No publication clause, but publication is mentioned. | X  No dispute resolution clause (or any mention of dispute resolution). | X  No confidentiality clause (or any mention of confidentiality). | ✓  No specific liability clause, but liability is mentioned. | X  No general provisions (miscellaneous clause). | X  No governing law clause (mentions compliance clause with “applicable” laws but does not specify). |
| **Johns Hopkins University**  ***Data Transfer and Use Agreement*** | X  No introduction or definitions are provided, but there is space for party details. | ✓  No specific purpose clause, but purpose is mentioned. | ✓  No specific termination clause, but deals with termination. | ✓  No specific obligations clause, but the duties of the parties are provided. | X  No reporting or auditing clause. | X  No intellectual property clause (or any mention of intellectual property). | ✓  No ownership clause, but ownership is mentioned under the terms and conditions. | X  No licensing clause (or any mention of licensing). | ✓  No publication clause, but publication is mentioned. | X  No dispute resolution clause (or any mention of dispute resolution). | X  No confidentiality clause (or any mention of confidentiality). | ✓  No specific liability clause, but liability is mentioned. | X  No general provisions (miscellaneous clause). | X  No governing law clause (mentions compliance clause with “applicable” laws but does not specify). |
| **Kawartha Lakes OHT**  ***Data Sharing Agreement*** | ✓  Contains information about the parties as well as recitals and a definitions clause. | ✓  Contains a purpose clause. | ✓  Contains clauses on both term and termination. | ✓  Contains a clause on obligations of participants, as well as a post-termination PHI obligations clause. | ✓  Contains an audits clause. Reporting is mentioned under the Schedule. | X  No intellectual property clause (intellectual property is mentioned under alternative dispute resolution). | X  No ownership clause (or any mention of ownership). | X  No licensing clause (or any mention of licensing). | X  No clause on publication or attribution (or any mention of it). | ✓  Contains a clause on alternative dispute resolution. | ✓  Contains a clause on confidential information. | ✓  Contains a clause on limitation of liability. | X  No general provisions (miscellaneous) clause. | ✓  Contains a governing law clause. |
| **Health Data Coalition**  ***Data Sharing Agreement*** | ✓  Contains information about the parties as well as an introduction and a definitions clause. | ✓  No specific purpose clause, but the purpose is found in the introduction. | ✓  Contains clauses on both term and termination. | ✓  Contains a clause on responsibilities. | X  No auditing or reporting clause, but reporting is mentioned in terms of inquiries, complaints, and technical issues. | X  No intellectual property clause (or any mention of intellectual property). | X  No ownership clause (or any mention of ownership). | X  No licensing clause (a license is mentioned under general provisions). | ✓  Contains a publications clause. | X  No dispute resolution clause (disputes are mentioned under general provisions). | ✓  Contains a clause on confidentiality and privacy. | X  No limitation of liability clause (or any mention of liability). | ✓  Contains a general clause. | ✓  No governing law clause (mentioned under the definition clause). |
| **Clinical Study Data Request Consortium**  ***Data Sharing Agreement*** | ✓  Contains information about the parties as well as a background and a definitions clause. | ✓  No specific purpose clause, but the purpose is found in the background and the clause on data sharing. | ✓  Contains a term and termination clause. | ✓  No specific obligations clause, but the duties of the parties can be found in the data sharing clause (and throughout the agreement). | X  No auditing or reporting clause. | ✓  Contains a clause on intellectual property. | ✓  No ownership clause, but ownership is dealt with under the data sharing clause. | ✓  No licensing clause, but licensing is dealt with under the data sharing and intellectual property clauses. | ✓  Contains a publications clause. | ✓  No dispute resolution clause, but dispute resolution is mentioned under the governing law clause. | ✓  Contains a clause on confidentiality. | ✓  No liability clause, but liability is dealt with under the clause on data sharing. | X  No general provisions (miscellaneous) clause. | ✓  Contains a governing law clause. |
| **GREGoR Consortium**  ***Data Sharing Agreement*** | ✓  Contains a terms and definitions clause. Party information appears at the end of the document, along with a glossary of definitions. | ✓  No specific purpose clause, but the purpose is found in the terms and definitions clause. | ✓  No specific term and termination clause, but they are dealt with under the miscellaneous clause. | ✓  Contains a clause on data sharing responsibilities. | X  No reporting or auditing clause, but reporting is mentioned under the miscellaneous clause. | X  No intellectual property clause (or any mention of intellectual property). | X  No ownership clause, but ownership is mentioned under the disclaimer. | X  No licensing clause (or any mention of licensing). | X  No publication clause, but publication is mentioned under the miscellaneous clause. | X  No dispute resolution clause (or any mention of dispute resolution). | X  No confidentiality clause (but confidentiality is mentioned). | X  No liability clause, but liability is mentioned under the disclaimer. | ✓  Contains a miscellaneous clause. | X  No governing law clause. |
| **National Center for Advancing Translational Sciences**  ***Data Transfer Agreement*** | ✓  Contains an introduction and information about the parties, but no definitions clause. | ✓  No specific purpose clause, but the purpose is found in the terms and conditions. | ✓  No specific term and termination clause, but they are dealt with under the terms and conditions. | ✓  No specific obligations clause, but responsibilities are dealt with under the terms and conditions. | X  No reporting or auditing clause. | X  No intellectual property clause (or any mention of intellectual property). | X  No ownership clause (or any mention of ownership). | X  No licensing clause (or any mention of licensing). | ✓  No publication clause, but publication is mentioned under the terms and conditions. | X  No dispute resolution clause (or any mention of dispute resolution). | X  No confidentiality clause (but mention of a Certificate of Confidentiality). | X  No liability clause, but liability is mentioned under the terms and conditions. | X  No general provisions (miscellaneous clause). | X  No governing law clause (mentions compliance clause with “applicable” laws). |
| **Bristol Myers Squibb**  ***Data Sharing Agreement*** | ✓  Contains information about the parties, preliminary statements, and a definitions clause. | ✓  No specific purpose clause, but the purpose is found in the introduction. | ✓  Contains a clause on term and termination. | X  No specific clause on obligations. | X  No reporting or auditing clause (or any mention of it). | ✓  Intellectual property is dealt with under the clause on inventions. | ✓  Ownership is dealt with under the clause on inventions. | ✓  Licensing is dealt with under the clause on inventions. | ✓  Contains a clause on publication. | ✓  No specific clause on dispute resolution, but disputes are dealt with under the governing law clause. | ✓  Contains a clause on confidentiality. | X  No liability clause (or any mention of liability). | ✓  Contains a miscellaneous clause. | ✓  No governing law clause (mentioned under the miscellaneous clause). |
| **Fred Hutch**  ***Multi-Party Data and Material Transfer Agreement*** | ✓  Contains an introduction, recitals, and a definitions clause. There is no specific information on the parties. | ✓  Contains a purpose within the introduction. | ✓  Contains a clause on term and termination. | ✓  Contains a clause on parties’ obligations. | ✓  Audits are dealt with under the compliance clause. No mention of reporting. | ✓  Contains a clause on intellectual property. | ✓  Ownership is dealt with under the clause on intellectual property. | ✓  Licensing is dealt with under the clause on intellectual property. | ✓  Contains a clause on publication. | ✓  No specific clause on dispute resolution, but disputes are dealt with under the law clause. | ✓  Contains a clause on confidentiality. | ✓  No liability clause, but liability is dealt with under the clause on indemnification. | X  No general provisions (miscellaneous clause). | ✓  Contains a governing law clause. Titled “Law” |
| **Department of Health Western Australia**  ***Material and Data Transfer Agreement*** | ✓  Contains information about the parties, a background, and a definitions clause. | ✓  Contains a clause on purpose in the schedule. | ✓  Contains clauses on both term and termination. | ✓  No specific obligation clause, but obligations are mentioned throughout. | X  No reporting or auditing clause. | ✓  Contains a clause on property and intellectual property. | ✓  Ownership is dealt with under the clause on property and intellectual property. | ✓  Licensing is dealt with under the clause on property and intellectual property. | ✓  Contains a clause on publications and publicity. | ✓  No specific clause on dispute resolution, but disputes are dealt with under the general clause. | ✓  Contains a clause on confidentiality. | ✓  Contains a clause on insurance, liability, and indemnity. | ✓  Contains a general provisions clause. | ✓  No governing law clause (mentioned under the general provisions clause). |
| **University of Newcastle**  ***Data Transfer Agreement*** | ✓  Contains information about the parties, a background, and a clause on the provision of data. | ✓  Contains a purpose clause. | ✓  Contains a termination clause. | ✓  General obligations are dealt with under the confidential information and privacy clause. | ✓  Reports are dealt with under the provision of information clause. | ✓  Contains a clause on intellectual property. | ✓  Ownership is dealt with under the clause on intellectual property. | ✓  Licensing is dealt with under the clause on intellectual property. | ✓  Contains a clause on publication. | X  No dispute resolution clause (or any mention of dispute resolution). | ✓  Contains a clause on confidential information and privacy. | ✓  No liability clause, but liability is dealt with under the clauses on no warranty and indemnities | ✓  Contains a general provisions clause. | ✓  No governing law clause (mentioned under the general provisions clause). |
| **ONDC**  ***Data Sharing Agreement*** | ✓  Contains an introduction and information about the parties, but no definitions clause. | ✓  Contains a purpose clause. | X  No term or termination clause. | X  No clause on obligations. | X  No reporting or auditing clause. | X  No intellectual property clause (or any mention of intellectual property). | X  No ownership clause (or any mention of ownership). | X  No licensing clause (or any mention of licensing). | ✓  Publication is dealt with under the outputs clause. | X  No dispute resolution clause (or any mention of dispute resolution). | X  No confidentiality clause. | X  No liability clause (or any mention of liability). | X  No general provisions (miscellaneous clause). | X  No governing law clause. |
| **Growing Up in New Zealand**  ***Data Access Agreement*** | ✓  Contains an introduction, information about the parties, and a definitions clause. | X  No specific purpose clause. | ✓  Contains a termination clause. | ✓  Contains a clause on institution obligations. | ✓  Contains a clause on audits. | ✓  Contains a clause on intellectual property. | X  No ownership clause (or any mention of ownership). | X  No licensing clause (or any mention of licensing). | ✓  Publication is dealt with under the outputs clause. | ✓  Contains a clause on disputes. | X  No confidentiality clause (or any mention of confidentiality). | ✓  Contains a liability clause. | ✓  Contains a general provisions clause. | ✓  No governing law clause (mentioned under the general provisions clause). |
| **Indian Society of Critical Care Medicine**  ***Data Transfer Agreement*** | ✓  Contains information on the parties, a recital, and a definitions clause. | ✓  Contains a purpose clause. | ✓  Contains a clause on term and termination of agreement. | X  No clause on obligations. | ✓  No specific reporting clause, but reporting is mentioned under the clause on representations, warranties, and covenants | ✓  Contains a clause on intellectual property and publication. | ✓  Contains a clause on data ownership. | ✓  Licensing is dealt with under the clause on intellectual property and publication. | ✓  Contains a clause on intellectual property and publication. | ✓  Disputes are mentioned under the governing law clause. | ✓  Contains a clause on confidentiality. | ✓  Contains a liability clause. | ✓  Contains a miscellaneous clause. | ✓  Contains a governing law clause. |
| **Dkfz German Cancer Research Center**  ***Data Transfer Agreement of Human Data for Research Purposes*** | ✓  Contains information on the parties, a preamble, and a definitions clause. | ✓  Contains a clause on purpose of the project. | ✓  Contains a clause on violation/termination of agreement. | ✓  No clause on obligations, but obligations are mentioned under the clause on data protection. | X  No clause on auditing or reporting (or any mention of it). | ✓  Contains a clause on intellectual property. | ✓  Ownership is dealt with under the clause on intellectual property. | ✓  Licensing is dealt with under the clause on intellectual property. | ✓  Contains a publications clause. | ✓  Disputes are mentioned under the governing law clause. | ✓  Contains a clause on confidentiality. | ✓  Liability is mentioned under the clause on legal statement. | X  No general provisions (miscellaneous) clause. | ✓  Contains a governing law clause. |
| **National Health Service (NHS) England**  ***Template Data Sharing Agreement*** | ✓  Contains information on the parties, and a definitions clause in the annex. | ✓  Contains a clause on purpose, objectives of the information sharing | ✓  Contains a clause on termination. | ✓  No specific obligation clause, but obligations are mentioned throughout. | ✓  No specific clause, but reporting is mentioned. | X  No intellectual property clause (or any mention of intellectual property). | X  No ownership clause (or any mention of ownership). | X  No licensing clause (or any mention of licensing). | X  No attribution or publication clause (or any mention of it). | ✓  Contains a dispute resolution clause. | X  No confidentiality clause (but confidentiality is mentioned throughout). | ✓  Liability is mentioned under the clause on remedies and no waiver. | ✓  Contains a general provisions clause. | ✓  No governing law clause (mentioned under the general provisions clause). |
| **Information Commissioner’s Office**  ***International Data Transfer Agreement*** | ✓  Contains a table for parties and signatures, information to assist in understanding, explanation, and a legal glossary at the end of the agreement | ✓  Contains a purpose clause. | ✓  Contains a clause on term, but no clause on termination. | ✓  Contains clauses on importer and exporter obligations. | ✓  No specific audit clause, but audits are mentioned under the clause on general importer obligations. No mention of reporting. | X  No intellectual property clause (or any mention of intellectual property). | X  No ownership clause (or any mention of ownership). | X  No licensing clause (or any mention of licensing). | X  No attribution or publication clause (or any mention of it). | ✓  No dispute resolution clause, but disputes are mentioned under the arbitration clause. | X  No confidentiality clause (or any mention of confidentiality). | ✓  Contains a liability clause (and liability is mentioned elsewhere). | ✓  Contains a general provisions clause. | ✓  Contains a governing law clause. |
| **Utrecht University**  ***Data Transfer Agreement*** | ✓  Contains information about the parties and a definitions clause. | ✓  No specific purpose clause, but purpose is mentioned under the use clause. | ✓  Contains an amendment, extension, and termination clause, as well as a term of agreement clause. | ✓  No specific obligations clause, but the use clause can be seen to cover obligations. | X  No clause on auditing or reporting (or any mention of it). | ✓  Contains a clause on intellectual property rights and ownership. | ✓  Contains a clause on intellectual property rights and ownership. | X  No licensing clause (or any mention of licensing). | ✓  Contains a publications clause. | ✓  No dispute resolution clause, but disputes are mentioned under miscellaneous. | ✓  Contains a confidentiality clause. | ✓  Contains a clause on warranties and liabilities. | ✓  Contains a miscellaneous clause. | ✓  No governing law clause (mentioned under the miscellaneous clause). |
| **Swiss Personalised Health Network**  ***Data Transfer and Use Agreement*** | ✓  Contains information about the parties, recitals, and a definitions clause. | ✓  Contains a purpose clause. | ✓  Contains clauses on both term and termination. | ✓  No specific obligation clause, but obligations are mentioned throughout. | ✓  Reporting is mentioned under the clause on data processing. | ✓  Contains a clause on intellectual property rights. | ✓  No specific ownership clause, but ownership is mentioned under the clauses on data processing and intellectual property rights. | ✓  No specific licensing clause, but licensing is mentioned under the clauses on data processing and intellectual property rights. | ✓  Contains a publications clause. | ✓  No dispute resolution clause, but disputes are mentioned under the governing law and jurisdiction clause. | ✓  Contains a confidentiality clause. | ✓  Contains a clause on liability, indemnification and third-party rights. | ✓  Contains a miscellaneous clause. | ✓  Contains a governing law clause. |
| **National Institute for Medical Research**  ***Data Transfer Agreement for Researchers/Organizations*** | ✓  Contains information about the parties, recitals, and a definitions clause. | ✓  No specific purpose clause, but purpose is mentioned under the recitals. | ✓  Contains a termination clause. | ✓  Contains clauses on the obligations of the provider and recipient. | X  No clause on auditing or reporting. | ✓  No specific intellectual property clause, but intellectual property is mentioned under the clause on legal title to data transferred and benefit sharing. | ✓  No specific ownership clause, but ownership is mentioned under the clause on legal title to data transferred and benefit sharing. | ✓  Contains clauses on permits, licenses, and approvals as well as non-exclusive licenses. | ✓  No specific publication clause, but publications are dealt with under the clause on obligation of the recipient. | ✓  No dispute resolution clause, but disputes are mentioned under the applicable law clause. | X  No confidentiality clause. | ✓  No liability clause, but liability is mentioned under the clauses dealing with the obligations of the provider and recipient. | X  No General provisions (miscellaneous) clause. | ✓  Contains a governing law clause. Titled “applicable law and severability”. |
